# Spin-Steered Photosynthesis of H_2_O_2_ in Magnetic Single-Atom Modified Covalent Triazine Frameworks: A Density Functional Theory Study

**DOI:** 10.3390/molecules29081840

**Published:** 2024-04-18

**Authors:** Feng Liao, Zhao Lu, Zhongliao Wang

**Affiliations:** 1 HKUST Shenzhen-Hong Kong Collaborative Innovation Research Institute, Shenzhen 515100, China; yb87402@umac.mo (F.L.); zhaolu@ust.hk (Z.L.); 2 Shenzhen Academy of Disaster Prevention and Reduction, Shenzhen 515100, China; 3 Research and Development Center, Shenzhen Foundation Engineering Co., Ltd., Shenzhen 515100, China; 4 Anhui Province Industrial Generic Technology Research Center for Alumics Materials, Huaibei Normal University, Huaibei 235000, China; 5 Anhui Province Key Laboratory of Pollutant Sensitive Materials and Environmental Remediation, Huaibei Normal University, Huaibei 235000, China; 6 School of Physics and Electronic Information, Huaibei Normal University, Huaibei 235000, China

**Keywords:** spin-steered, photosynthesis of H_2_O_2_, magnetic single atom, CTFs, DFT

## Abstract

Covalent Organic Frameworks (COFs) demonstrate promising potential in the photocatalytic synthesis of H_2_O_2_ owing to favorable light absorption, superior charge separation, and considerable surface area. However, the efficiency of H_2_O_2_ photosynthesis is impeded by insufficient O_2_ adsorption sites and a high reaction barrier. In this work, various metal single atoms (Fe, Co, Ni) are introduced onto covalent triazine frameworks (CTFs) with N-N coordination sites to significantly enhance O_2_ adsorption and optimize H_2_O_2_ synthesis. Computational findings suggest that the presence of Fe, Co, and Ni not only enhances O_2_ adsorption but also exerts an influence on the reaction pathway of H_2_O_2_. Significantly, Fe exhibits a distinct advantage in modulating O_2_ adsorption through its unique electron spin state when compared to Co and Ni, as confirmed by crystal orbital Hamilton population (COHP) analysis. Additionally, this integration of metal atoms also improves light absorption and charge separation in CTFs. The study provides strategic insight into elevating H_2_O_2_ production by incorporating tailored metal single atoms into COFs.

## 1. Introduction

Photocatalytic synthesis of hydrogen peroxide (H_2_O_2_) holds significant value in fields such as healthcare, environmental remediation, food processing, and chemical synthesis [1]. Traditional inorganic semiconductors face limitations in H_2_O_2_ synthesis efficiency due to severe charge recombination and complex surface structures that hinder the elucidation of the mechanism underlying H_2_O_2_ generation [2]. Covalent Organic Frameworks (COFs), with their well-defined structures and efficient charge separation, not only demonstrate superior catalytic efficiency in H_2_O_2_ production experimentally but also facilitate the theoretical elucidation of the catalytic mechanism [3,4,5]. However, COFs, typically composed of light non-metal elements, exhibit a high barrier for O_2_ adsorption and activation, limiting their catalytic performance. Therefore, it is essential to advance the O_2_ adsorption capability of COFs to elevate the efficacy of H_2_O_2_ production [6,7].

Single-atom catalysts, renowned for their exceptional atomic utilization, remarkable catalytic activity, and distinct active sites, have emerged as a focal point in the modification of photocatalysts [8,9,10]. However, the experimental preparation and exploration of single-atom catalysts loaded within COFs present formidable challenges in terms of cost and complexity, thereby impeding progress in H_2_O_2_ photosynthesis [11,12,13]. Theoretical investigations into the utilization of single-atom-loaded COFs for photocatalytic H_2_O_2_ synthesis remain highly significant in screening exceptional catalysts. Among various single atoms, magnetic Fe, Co, and Ni demonstrate promising H_2_O_2_ activity due to their enhanced interaction with oxygen [14]. However, the stability of these magnetic single atoms relies on robust coordination environments, necessitating the design of COFs with sufficient coordination capabilities [15,16]. However, detailed theoretical insights into how magnetic single atoms, especially their spin electronics, modulate the H_2_O_2_ reaction are notably absent [17]. Thereby, further exploration is needed to understand the catalytic mechanisms of these magnetic single atoms in H_2_O_2_ synthesis, particularly the role of spin electronics in O_2_ adsorption and activation.

Motivated by this, we designed and investigated the impact of covalent triazine frameworks (CTFs) with N-N coordination sites loaded with magnetic single atoms of Fe, Co, and Ni on the photocatalytic synthesis of H_2_O_2_. The structure, optical properties, electronic structure, charge separation and transfer, and the reaction pathways of CTFs loaded with magnetic single atoms were systematically simulated and investigated. The impact of the electronic spin states of Fe, Co, and Ni single atoms on the adsorption and activation capabilities of O_2_ was profoundly assessed based on density of states analysis and crystal orbital Hamilton population (COHP) calculations. This work aims to provide a reference for the experimental design of highly active magnetic single-atom photocatalysts for H_2_O_2_ synthesis.

## 2. Results and Discussion

**Figure 1:**
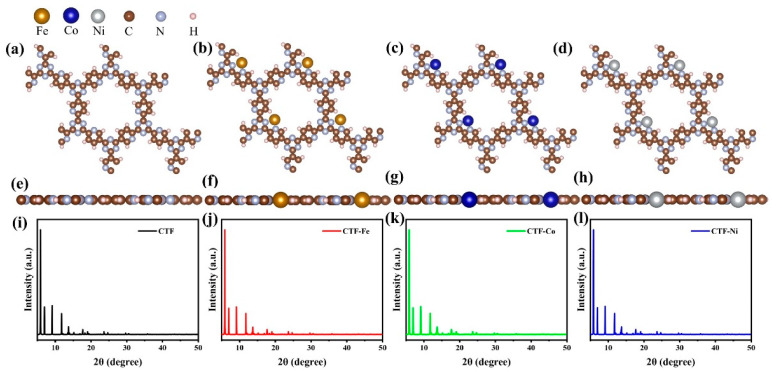
Top perspective of crystal structure, (**a**) CTF, (**b**) CTF–Fe, (**c**) CTF–Co, and (**d**) CTF–Ni. Crystal structure side view of (**e**) CTF, (**f**) CTF–Fe, (**g**) CTF–Co, and (**h**) CTF–Ni. Simulated XRD pattern of (**i**) CTF, (**j**) CTF–Fe, (**k**) CTF–Co, and (**l**) CTF–Ni.

A CTF with alternating connections of TR and pyridine was constructed for investigating the mechanism of different single-atom loadings on the photocatalytic performance of CTFs (Figure 1a). The monolayer CTF consists of 21 C atoms, 9 N atoms, and 9 H atoms. The positions of single-atom metals are located between TR and pyridine. The single atoms form chemical bonds with the N atoms of the TR unit and the N atoms of the pyridine unit to be fixed between TR and pyridine. Fe, Co, and Ni from the same period were selected as single-atom metals. The atomic radii of Fe, Co, and Ni are 117, 116, and 115 pm, respectively. The similarity in atomic radii enables Fe, Co, and Ni to be loaded onto the unit sub-sites in a consistent manner, thereby avoiding the introduction of additional variables arising from significant structural differences in the optimized configuration of CTFs loaded with different single atoms. This ensures that the mechanism underlying various single-atom loadings on the photocatalytic performance of CTFs remains unchanged. The pore size of the single-atom-loaded CTF is basically consistent with that of our CTF, so the strategy of selecting single-atom loading to modify our CTF does not reduce the advantages of the CTF in terms of specific surface area.

Since the matrix materials are all CTFs, the X-ray diffraction (XRD) patterns of the CTFs loaded with three different single atoms are basically the same (Figure 1i–l). The three strongest peaks of the four materials are all before 10°, at 5.9, 7, and 9.15°, respectively. This distribution of the three strongest peaks is due to the regular pore structure and highly ordered arrangement of CTFs, as well as the short distance between structural units of the materials. Similarly, the loading of single atoms does not change the lattice parameters of CTF (a = b = 14.574 Å, c = 15 Å, α = β = 90°, γ = 120°), which belong to the typical hexagonal crystal system. This is dictated by the positioning of the single-atom loading site, which resides within the planar ring of the single-atom-loaded CTF rather than the interlayer.

An analysis of bond lengths and bond angles at the sites where single atoms are loaded onto the four CTFs was conducted to evaluate the influence of different single-atom loadings on the CTF structure. Firstly, the bond lengths of C–N in TR inside the CTF are 1.349 and 1.344 Å, both exceeding the bond length of the isolated C–N bond in TR (Table 1). This is because pyridine interacts with TR, attracting TR towards pyridine. The loading of single atoms has a significant impact on the bond lengths of TR and pyridine. When a single atom is present between TR and pyridine, the C1–N2 and N2–C3 bonds inside TR are both elongated (to 1.383 and 1.391 Å, respectively). Similarly, pyridine is also attracted by the Fe atom, elongating the N5–C6 bond to 1.343 Å. The structural changes in CTF–Co and CTF–Ni are consistent with those in CTF–Fe. Additionally, the bond lengths of Fe–N, Co–N, and Ni–N gradually decrease to 2.153, 1.981, and 1.934 Å, respectively.

**Figure 2:**
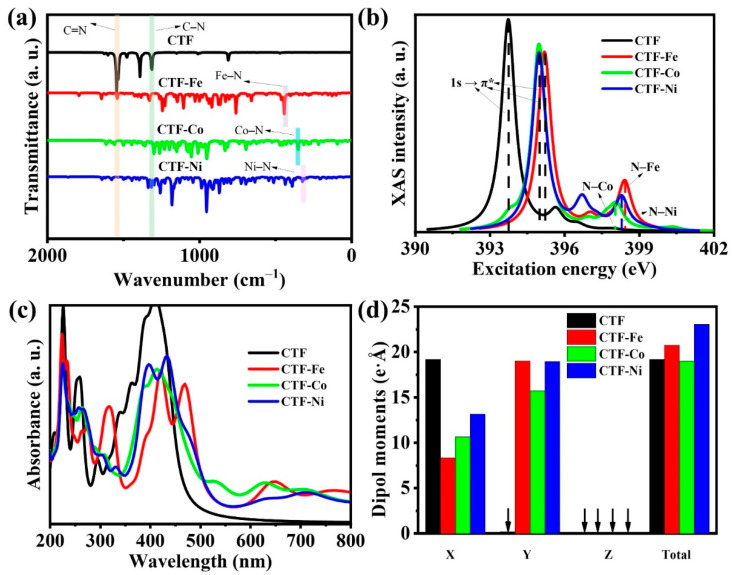
(**a**) Simulated IR spectra, (**b**) synchrotron XAS of the N K edge, (**c**) UV-Vis spectrum, and (**d**) dipole moments on different components of CTF, CTF–Fe, CTF–Co, and CTF–Ni.

Infrared spectroscopy (IR) is widely recognized for its ability to capture molecular vibrations and rotations, such as bending, stretching, and twisting, which are pivotal in identifying functional groups within materials. For CTFs, the infrared characteristic absorption peaks of TR are located at 1315 and 1545 cm^−1^ (Figure 2a). The absorption peak at 1315 cm^−1^ corresponds to the stretching vibration mode of the C–N bond on TR, while the peak at 1545 cm^−1^ represents the stretching vibration of the carbon–nitrogen double bond. For the three CTFs with single-atom loadings, their characteristic peaks belonging to TR are weakened to varying degrees. This is because the loading of single atoms restricts the stretching vibrations of the carbon–nitrogen single bond and double bond. The three CTFs loaded with single atoms exhibit characteristic absorption peaks at low wave numbers that are not present in ordinary CTFs. The infrared absorption peak at 437 cm^−1^ in CTF–Fe corresponds to the bending vibration of the Fe–N bond. In CTF–Co and CTF–Ni, the bending vibration peaks of the Co–N and Ni–N bonds are located at 348 and 318 cm^−1^, respectively.

Performing X-ray absorption spectroscopy (XAS) calculations on CTFs can help understand the energy levels of the inner-shell electrons of CTF atoms, providing information on the electronic states and chemical environments of specific elements in the material. Because both TR and pyridine contain N with lone pair electrons, CTF can form good coordination with metal single atoms through N. Therefore, the energy range of XAS simulation focuses on the excitation energy of N (393–409 eV) to elucidate the different coordination situations of different metals with N. It is well known that the 1s orbital of N is an inner-shell orbital, while the π orbital of N is the antibonding orbital related to the π bond when N is combined with other elements. When the energy of the incident X-ray matches the electron transition from the N 1s orbital to the π* orbital, an XAS absorption edge occurs (Figure 2b). The characteristic peak of CTF for 1s → π* is located at 393.71 eV, and the peak shifts towards higher energy with the loading of single atoms. This indicates that when the coordination environment of nitrogen atoms changes, the chemical bond properties around them also change. Among them, CTF–Fe shows a new characteristic peak at 398.45 eV (not present in CTF), which should be attributed to the N–Fe bond. Meanwhile, CTF–Co and CTF–Ni have characteristic peaks at 398.01 and 398.28 eV, respectively, reflecting the bonding between the metal atom and N.

DFT calculations reveal that CTFs predominantly absorb shorter UV wavelengths, particularly between 200–278 nm and 309–467 nm, showing limited visible-light absorption. Notably, single-atom doping extends CTF’s absorption edge into longer wavelengths of 530 nm for CTF–Fe, 521 nm for CTF–Co, and 540 nm for CTF–Ni—thereby enhancing absorption in the visible spectrum. This enhancement is attributed to the metal single atoms inducing charge transfer on the CTF surface, creating localized charge polarization zones. These zones alter the light absorption characteristics of the carbon materials, improving visible light uptake. Additionally, metal doping reconfigures CTF’s energy levels, introducing new levels or modifying existing ones within the visible range, thereby amplifying visible light absorption. The introduction of metal single atoms may also trigger localized surface plasmon resonance effects, strengthening light-material interactions and boosting absorption. Overall, metal single atom incorporation significantly augments CTFs’ light absorption capabilities, offering a strategic avenue for enhancing photocatalytic performance.

Charge separation stems from an internal electric field due to uneven charge distribution, quantified by the dipole moment in molecules or crystals. DFT calculations of the dipole moments for CTF and its single-atom variants reveal how single-atom modification impacts CTF’s internal electric field. Initially, CTF exhibits a dipole moment predominantly in the X direction, valued at 19.19 e·Å. Single-atom doping alters this distribution significantly, decreasing the dipole moment in the X direction while markedly increasing it in the Y direction. This change suggests that the addition of a single atom introduces symmetrical structures or electronic distributions in the X direction, equalizing the charge distribution and thus reducing the dipole moment. Conversely, in the Y direction, single-atom doping creates asymmetric electronic distributions or charge densities, enhancing the dipole moment. Among the variants, CTF–Ni demonstrates the highest dipole moment, indicating the strongest internal electric field and the greatest potential for photogenerated charge carrier separation. This analysis underscores the profound effect of single-atom doping on enhancing photocatalytic efficiency by modulating their internal electric fields and charge separation capabilities.

**Figure 3:**
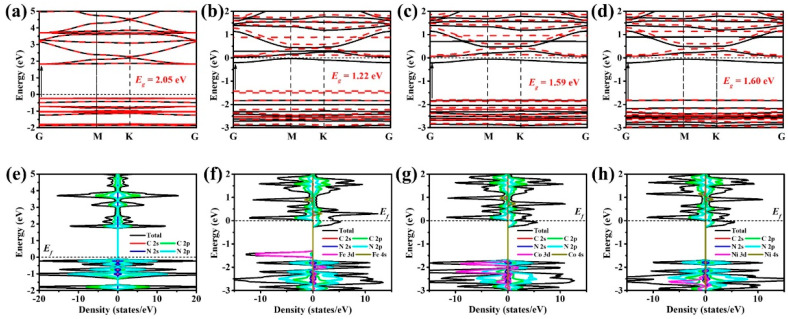
Band structure of (**a**) CTF, (**b**) CTF–Fe, (**c**) CTF–Co, and (**d**) CTF–Ni. Density of states (DOS) of (**e**) CTF, (**f**) CTF–Fe, (**g**) CTF–Co, and (**h**) CTF–Ni.

The band structure reveals that CTF exhibits direct bandgap semiconductor behavior, characterized by a well-suited bandgap (E_g_) of 2.05 eV, rendering it highly suitable for efficient photocatalytic H_2_O_2_ production (Figure 3a). The VBM of CTF primarily consists of the 2p levels of carbon and nitrogen, with a more significant contribution from nitrogen due to its lower 2p orbital energy level (Figure 3e). The band gaps are significantly underestimated by the PBE functional due to the presence of a delocalization error, particularly for d-band metals. Therefore, the incorporation of Hubbard U was employed as a corrective measure to rectify the calculated band gap values. Introducing single atoms into CTF alters its band structure, affecting the bandgap values for CTF–Fe (1.22 eV), CTF–Co (1.59 eV), and CTF–Ni (1.60 eV) without changing their direct bandgap nature (Figure 3b–d). This modification shifts the Fermi level into the conduction band minimum (CBM), rendering it an n-type degenerate semiconductor, which improves electron transport and catalytic efficiency due to its metallic-like properties. For CTF–Fe, the VBM is predominantly formed by spin-down 3d orbitals of Fe, contributing to its magnetic properties and the smallest band gap among the modified CTFs (Figure 3f). The VBM of CTF–Co shifts to more negative energy levels, resulting in an increased band gap compared to that of CTF–Fe (Figure 3g). These separated energy bands in VBM are mainly contributed by magnetic single atoms. Due to the separation in space of these single atomic orbitals, it results in insufficient splitting of the orbitals into continuous energy bands. Despite the asymmetric distribution of spin states, the disparity between spin-up and spin-down states in CTF–Co VBM is negligible. The CTF–Ni VBM closely resembles the overall density of states (DOS), as the Ni spin-down 3d orbitals are deeply integrated into the valence band, thereby having minimal impact on VBM construction (Figure 3h).

**Figure 4:**
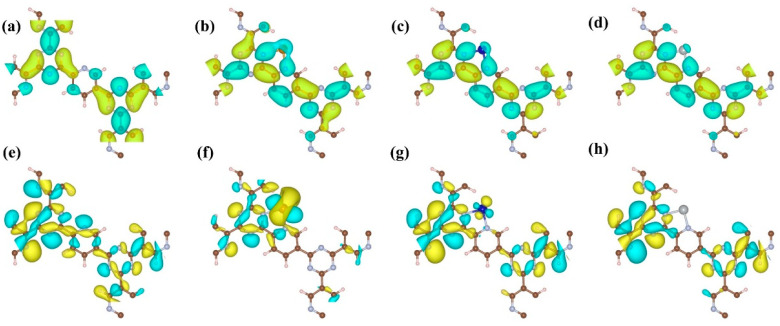
Top view of the CBM of (**a**) CTF, (**b**) CTF–Fe, (**c**) CTF–Co, and (**d**) CTF–Ni and the VBM of (**e**) CTF, (**f**) CTF–Fe, (**g**) CTF–Co, and (**h**) CTF–Ni.

**Figure 5:**
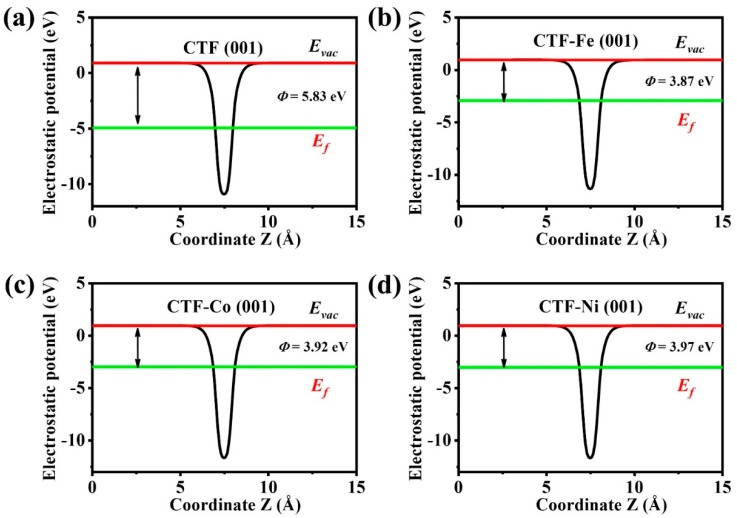
Electrostatic potentials of the (**a**) CTF (001) surface, (**b**) CTF–Fe (001) surface, (**c**) CTF–Co (001) surface, and (**d**) CTF–Ni (001) surface.

To elucidate the impact of single-atom doping on CTF’s electronic structure more vividly, DFT calculations were utilized to map the distribution of VBM and CBM energy levels across different atoms (Figure 4). For the undoped CTF, VBM and CBM energy levels are evenly spread across all atoms, mirroring the density of states (DOS) and potentially facilitating in situ recombination of photoexcited charge carriers. However, doping CTF with single atoms markedly alters these distributions. In CTF–Fe, the VBM prominently features on Fe sites, aligning with its DOS profile, indicating a significant reconfiguration of energy levels towards Fe atoms. Similarly, in CTF–Co, the 3d energy levels of Co partially constitute the VBM, as revealed in its 2D distribution plot, though Co atoms constitute only a fraction of the VBM and CBM. In contrast, the VBM distribution in CTF–Ni is minimally affected by Ni atoms due to their deep 3d energy levels, which are too distant to exert any influence on the VBM. Furthermore, the work function of CTF is notably influenced by single-atom doping, as evidenced by a reduced work function of 3.87, 3.92, and 3.97 eV for CTF–Fe, CTF–Co, and CTF–Ni compared to the original value of 5.83 eV (Figure 5). This reduction is attributed to donor doping from metal single atoms, elevating the Fermi level and consequently lowering the work function of CTF, highlighting the profound effect of single-atom incorporation on the electronic properties.

**Figure 6:**
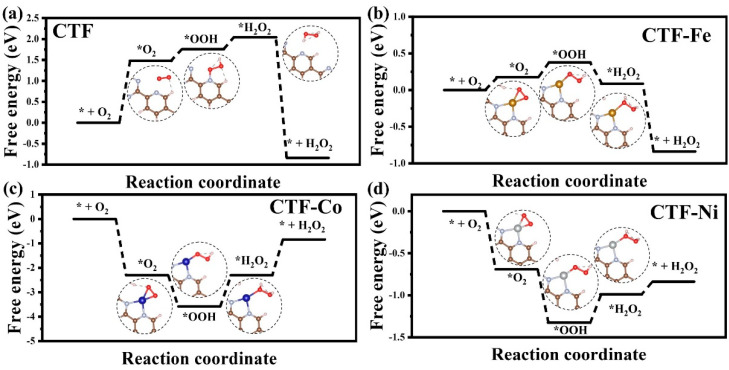
Free energy diagrams for the reduction of O_2_ to H_2_O_2_ for (**a**) CTF, (**b**) CTF–Fe, (**c**) CTF–Co, and (**d**) CTF–Ni.

To investigate the influence of different single-atom loadings on the mechanism of CTF photocatalytic production of H_2_O_2_, the Gibbs free energy of four CTF reaction pathways was calculated. The rate-determining step for CTF is the adsorption of O_2_ on pyridine by forming a hydrogen bond with H (* + O_2_ = *O_2_), with a specific value of 1.48 eV (Figure 6). Single-atom loading can effectively reduce the reaction barrier of CTF for the reduction of O_2_ to produce H_2_O_2_. The reaction barriers for CTF–Fe, CTF–Co, and CTF–Ni are 0.2 eV (*O_2_ + H^+^ + e = *OOH), 1.46 eV (*H_2_O_2_ = * + H_2_O_2_), and 0.32 eV (*OOH + H^+^ + e = * H_2_O_2_), respectively. The introduction of single-atom sites directly changes the adsorption site of O_2_, shifting it from N on pyridine to the single-atom metal. Initially, O_2_ is not inclined to adsorb on the surface of CTF, so the maximum adsorption barrier for CTF is 1.48 eV. However, when the adsorption site of O_2_ changes to the metal, the difficulty of O_2_ adsorption on the CTF surface drops sharply. The adsorption barrier of O_2_ on CTF–Fe is only 0.17 eV, while CTF–Co and CTF–Ni can even adsorb O_2_ spontaneously. Among them, the ability of CTF–Co to adsorb oxygen is much higher than the other three materials. The electron structure of CTF determines the difficulty of CTF adsorbing O_2_. The loading of metal single atoms makes CTF an n-type degenerate semiconductor, allowing more electrons to transfer to O_2_ to increase the coupling ability with oxygen. Although CTF–Co and CTF–Ni have stronger O_2_ adsorption capabilities than CTF–Fe, it does not mean that CTF–Co and CTF–Ni are superior to CTF–Fe in the entire process of H_2_O_2_ production. The rate-determining step for CTF–Fe is only 0.2 eV, which is much smaller than CTF–Co and Ni. For CTF–Co, its excessive adsorption of O_2_ leads to a high energy requirement to desorb the generated H_2_O_2_ from the metal site. Similarly, due to the strong adsorption of O_2_ on CTF–Ni, *OOH on CTF–Ni is difficult to convert to *H_2_O_2_ through hydrogenation. In conclusion, the loading of metal single atoms can effectively reduce the reaction barrier of CTF for the reduction of O_2_ to produce H_2_O_2_, and the unique electron structure of CTF–Fe achieves a balance between O_2_ adsorption and desorption, minimizing the reaction barrier.

**Figure 7:**
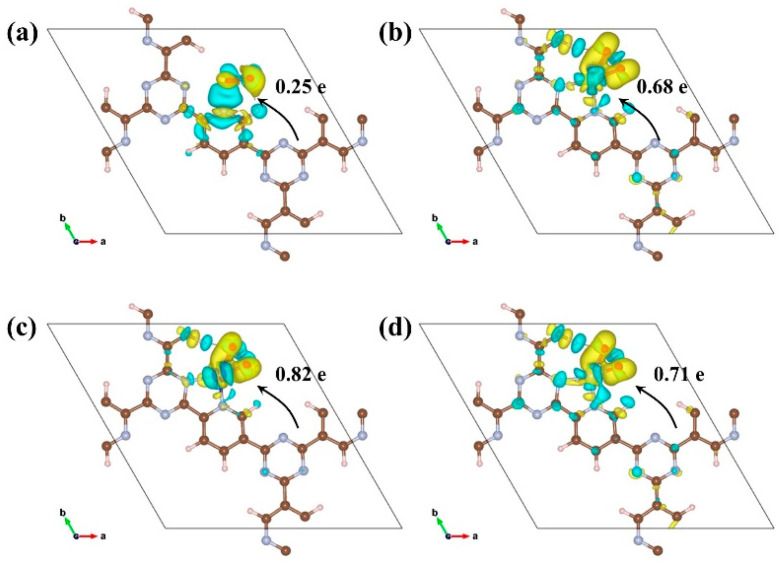
Top view of the charge density difference of (**a**) CTF, (**b**) CTF–Fe, (**c**) CTF–Co, and (**d**) CTF–Ni adsorbed with O_2_. (Increased charge in yellow, decreased charge in cyan).

Simulations of the differential charge density and Bader charges between CTF and adsorbed O_2_ were conducted to elucidate the direction and magnitude of charge transfer between them. Initially, all four CTFs were found to donate electrons during O_2_ adsorption. The minimum electron transfer from CTF to O_2_ was 0.25 e, while CTF–Co exhibited the maximum at 0.82 e (Figure 7). CTF–Ni closely followed with 0.71 e transferred to O_2_. The quantity and direction of electron transfer correspond to the trend in the Gibbs free energy change for O_2_ adsorption. Upon the adsorption of oxygen molecules on a catalyst surface, the formation of strong chemical bonds between the oxygen molecule and the surface typically requires a significant amount of electron transfer to activate and adsorb the oxygen molecule. Insufficient electron transfer to the oxygen molecule can result in weaker bonding between the oxygen molecule and the surface, reducing the adsorption capability. Thus, a lower number of electrons transferred to the oxygen molecule is less favorable for its adsorption.

**Figure 8:**
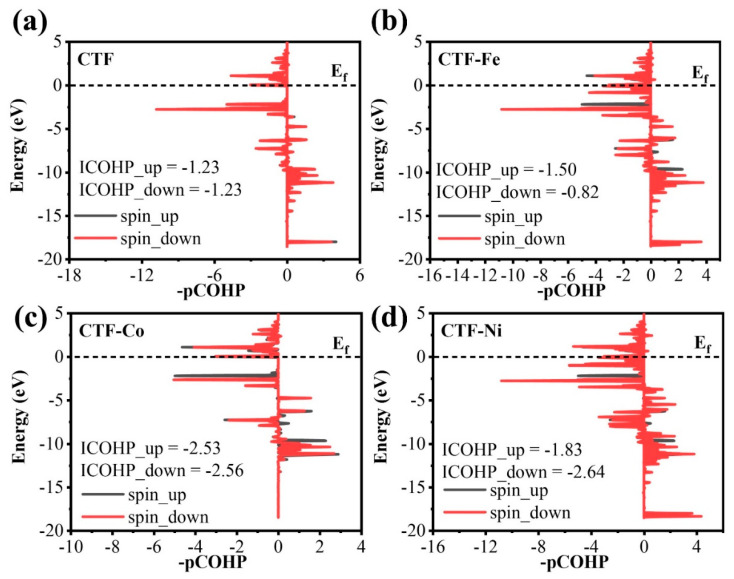
COHP of (**a**) CTF, (**b**) CTF–Fe, (**c**) CTF–Co, and (**d**) CTF–Ni.

To elucidate the correlation between O_2_ bonding strength to CTF and electron transfer, we carried out a COHP analysis on O_2_ adsorption for four different CTF configurations (Figure 8). COHP plots indicated stable bonds between CTF and O_2_ across all configurations, as evidenced by predominantly positive contributions. The bonding strength was quantitatively assessed by calculating the integrated COHP (ICOHP) values, revealing CTF exhibited the highest positive ICOHP at −2.46 eV, while CTF–Co displayed the strongest bond with O_2_, marked by the most negative value at −5.09 eV (Figure 8a,c). CTF–Ni and CTF–Fe had ICOHP values of −4.47 eV and −2.32 eV, respectively, illustrating that more negative values denote stronger bonds (Figure 8b,d). Thus, CTF–Co exhibited the highest affinity towards O_2_, while CTF–Fe demonstrated the lowest binding strength. Further analysis to discern orbital contributions to the bonding between O_2_ and CTF are shown in CTF, where the 2p orbital of O and the 2p orbital of N hybridize to form bonds, with spin-up and spin-down states contributing equally, at approximately −1.23 eV. CTF–Fe exhibits a contribution of −1.50 eV for spin-up states and −0.82 eV for spin-down states to the bonding of O_2_. This variance primarily stems from the interaction between the O 2p orbital and the Fe 3d orbital for the spin-up ICOHP and the O 2s orbital with the Fe 4s orbital for the spin-down ICOHP. For CTF–Ni, the spin-up state contribution of −1.83 eV mainly comes from the coupling between the O 2s orbital and the Ni 4p orbital, while the spin-down state contributes −2.64 eV, involving the coupling between the O 2p orbital and the Ni 3d orbital. CTF–Co shows consistent contributions from both spin states, chiefly arising from the coupling between the O 2s orbital and the Co 4p orbital and the O 2p orbital and the Co 3d orbital. This comprehensive analysis underscores that the spin asymmetry significantly influences the ICOHP value and, thus, the bonding strength when O_2_ adsorbs at single-atom sites.

## 3. Computational Details

Density Functional Theory (DFT) calculations were performed using the Vienna ab initio Simulation Package (VASP) with the projector augmented-wave (PAW) approach. The exchange–correlation potential was modeled using the generalized gradient approximation (GGA) framework, specifically the Perdew, Burke, and Ernzerhof (PBE) formulation [18]. To address the strong correlation effects in the Fe, Co, and Ni 3d orbitals, Hubbard U values were adjusted to 6.28, 6.32, and 7.67 eV, respectively [19]. Surface analyses were carried out with a Brillouin zone sampling mesh of 2 × 2 × 1 K-points, and the energy cutoff was set to 500 eV. Structural optimizations continued until the energy and force convergence thresholds were met, at less than 1 × 10^−5^ eV and 0.02 eV/Å, respectively. A vacuum layer of 15 Å was applied to minimize interactions between the periodic models of the surface. Van der Waals (vdW) forces were accounted for using the DFT-D3 method with zero damping by Grimme [20]. The Gibbs free energy (ΔG) was delineated as the electronic energy change (ΔE) and the zero-point energy change (ΔE_ZPE_), minus the product of temperature (T) and entropy change (ΔS), succinctly expressed as ΔG = ΔE + ΔE_ZPE_ − TΔS. The assessment of zero-point energies and the analysis of vibrational frequencies and entropies for gas-phase molecules were facilitated through the VASPKIT utility, leveraging frequency analysis [21]. Spectroscopic investigations, including assessments of vibrational and excited-state spectra, were carried out using the CP2K-2023.2 suite, utilizing the PBE functional for the representation of systems. Electronic structures were evaluated via unrestricted Kohn–Sham DFT within the Gaussian and Plane Waves (GPW) framework, incorporating Goedecker–Teter–Hutter (GTH) pseudopotentials and the triple-zeta valence plus two polarization functions (TZV2P-MOLOPT-GTH) as the basis set for all elements involved. The cutoff for plane waves, the convergence threshold for the self-consistent field (SCF), and the force convergence criteria were established at 400 Ry, 1 × 10^−5^ Hartree, and 4.5 × 10^−4^ Bohr per Hartree. Excited states analyses and their spectra were executed through the Multiwfn 3.8 software package [22].

## 4. Conclusions

In summary, CTFs equipped with N-N coordination sites are efficient platforms for crafting magnetic single-atom photocatalysts. Initially, the incorporation of magnetic metal single atoms as electron donors enhances the positioning of the Fermi level, resulting in a reduction in both the work function and bandgap. Consequently, this improvement facilitates the efficient capture and utilization of solar energy by CTFs. Additionally, the integration of magnetic metal single atoms enhances the surface polarization of CTFs, thereby effectively facilitating the transfer and separation of photoexcited charges. Additionally, the incorporation of magnetic metal single atoms significantly enhances the adsorption capacity of CTFs for O_2_ molecules, thereby substantially reducing the energy barrier associated with H_2_O_2_ synthesis. Interestingly, excessively strong O_2_ adsorption leads to difficulties in the conversion of intermediates and the desorption of H_2_O_2_, thereby raising the energy barrier for H_2_O_2_ formation. Among the magnetic elements Fe, Co, and Ni, Fe exhibits a weaker coupling between its 3d orbitals and the 2p orbitals of O_2_ molecules. Notably, the spin-down orbitals in the Fe 3d orbitals exhibit even weaker coupling with O 2p, resulting in weak adsorption capacity for O_2_. This spin-regulated adsorption mechanism optimizes the adsorption of O_2_, facilitates hydrogenation reduction, and enhances H_2_O_2_ desorption, thereby significantly reducing the energy barrier for H_2_O_2_ formation. This study offers valuable insights into spin-ameliorated H_2_O_2_ photosynthesis by magnetic single-atom modified CTFs.

## Figures and Tables

**Table 1:** Bond length of CTF, CTF–Fe, CTF–Co, and CTF–2Ni.

Bond Length (Å)	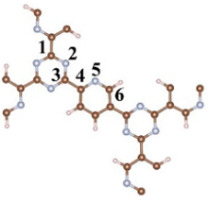	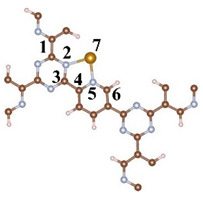	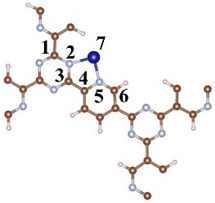	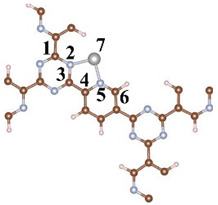
C1–N1	1.349	1.383	1.361	1.361
N1–C2	1.344	1.391	1.386	1.386
C2–C3	1.508	1.463	1.466	1.464
C3–N2	1.349	1.371	1.383	1.383
N2–C4	1.336	1.343	1.343	1.343
N1–Fe1	–	2.153	–	–
N1–Fe1	–	2.077	–	–
N1–Co1	–	–	1.981	–
N2–Co1	–	–	2.004	–
N1–Ni1	–	–	–	1.934
N1–Ni1	–	–	–	1.950

## Data Availability

Data are contained within the article.
